# A nested virtualization tool for information technology practical education

**DOI:** 10.1186/s40064-016-2041-8

**Published:** 2016-04-12

**Authors:** Carlos Pérez, Juan M. Orduña, Francisco R. Soriano

**Affiliations:** Departamento de Informática, Universidad de Valencia, Avda. Universidad, s/n, 46100 Burjassot, Valencia Spain; IRTIC, Universidad de Valencia, Polígono La Coma, s/n, Paterna, Valencia Spain

**Keywords:** Nested virtualization, Network security, Computer networks, Lecture-based learning, System administration, Problem-based learning

## Abstract

**Background:**

A common problem of some information technology courses is the difficulty of providing practical exercises. Although different approaches have been followed to solve this problem, it is still an open issue, specially in security and computer network courses.

**Results:**

This paper proposes NETinVM, a tool based on nested virtualization that includes a fully functional lab, comprising several computers and networks, in a single virtual machine. It also analyzes and evaluates how it has been used in different teaching environments.

**Conclusions:**

The results show that this tool makes it possible to perform demos, labs and practical exercises, greatly appreciated by the students, that would otherwise be unfeasible. Also, its portability allows to reproduce classroom activities, as well as the students’ autonomous work.

## Background

Security, system administration and computer networks are fundamental elements of information technology (IT) systems today, and many related courses (operating systems, computer network fundamentals, computer and network security, network management, etc.) are included in computer science graduate and postgraduate degrees. A common problem that arises in all these courses is the difficulty of designing practical exercises.

It is widely accepted that students learn more effectively from courses that provide for involvement in practical activities (e.g., setting up a customized network, installing and configuring network services, testing ethical hacking techniques, etc.), as shown in a wide variety of papers, conferences and books devoted to computer science education (Sarkar 2006; Trabelsi and Alketbi 2013; O’Grady 2012; Carter 2013). However, it is very difficult to design practical exercises that do not seriously affect the infrastructure where these exercises are done. Operating system administration exercises or penetration tests are examples of such activities, that may be even illegal. Simulation tools such as Packet Tracer from Cisco (2014) could be an alternative to real systems. However, the complexity of simulating real systems make these tools to focus on certain subsystems (i.e. the network), thus limiting their scope.

Virtualization techniques were proposed some years ago as an efficient alternative for teaching computer networks related courses in a secure and controlled environment (Bulbrook 2006; Gaspar et al. 2008; Pizzonia and Rimondini 2008; Burd et al. 2009), and they are currently used in many courses (Faircloth 2011; Salah 2014; Raman et al. 2014). These proposals use virtualization in order to set up network and computer infrastructures that resemble the actual ones (even in the user interface), while they provide the required security and isolation from the actual infrastructures. These tools provide users with an easily reproducible environment, and they allow students’ autonomous work. Virtualization and nested virtualization tools have also been proposed in many education environments (Bower 2010; Wannous et al. 2012).

Traditionally, two different approaches have been used: the first one is to provide copies of virtual machine images to the students so that they run them in its own computer, and the second one is to setup a virtual laboratory using the institution’s infrastructure, providing students with remote access. Both of these approaches present some inconveniences. The first one should be limited to a single virtual machine in order to provide ease of use. Otherwise, it requires that each student configures its own virtual lab using several images and creating its own virtual network infrastructure (a non-trivial and error-prone process, which is bounded by the resources of the host computer). The second approach requires significant investment in infrastructure resources, and the requirements are proportional to the number of students. Additionally, the availability of the resources cannot be guaranteed once the course finishes (for example, in subsequent years).

The advent of cloud computing and the increasing availability of web services during the last years (Marinescu 2013; Amazon 2014; Google 2014) has allowed to go one step further, and some cloud-based virtualization tools for online teaching have been proposed (Salah 2014; Willems et al. 2011; Abraham 2013; Xu et al. 2014). Nevertheless, the deployment of cloud services adds some drawbacks to virtualization tools. First, the use of a given cloud infrastructure forces the user to learn and use a concrete technology and services, making the course dependent on a given service provider. Second, the number of students in a given course may require a cloud infrastructure size that exceeds the maximum size that the provider offers for free, increasing the cost of the course. Third, the use of cloud resources may add significant latencies that affect the interactivity of the exercises. Finally, the reproducibility and usability along time is seriously affected, since students are not guaranteed that the cloud infrastructure is accessible some time after the course finishes (Son et al. 2012), like the second approach in the use of virtualization techniques described above.

In order to avoid the problems introduced by these approaches, this paper proposes NETinVM, a tool based on nested virtualization (virtual machines inside a virtual machine) that includes a fully functional lab in a single virtual machine. This lab comprises three interconnected networks with several computers attached to each network, providing a portable and realistic scenario for teaching courses related to security, system administration and computer networks. The paper analyzes the use of NETinVM in different learning techniques [Problem-Based Learning (PBL) and traditional Lecture-Based Learning (LBL)] applied to courses of different computer science fields. The results show that this tool allows to perform labs and practical exercises that would otherwise be unfeasible. Also, it allows to reproduce the results of the proposed exercises, providing portability and allowing the students to work autonomously.

The rest of the paper is organized as follows: “Implementation” section summarizes the implementation and main features of NETinVM. Next, “Results and discussion” section shows the application of NETinVM to different learning and training environments and the results obtained with this tool. Finally, “Conclusions” section shows come conclusion remarks and future work to be done.

## Implementation

**Fig. 1:**
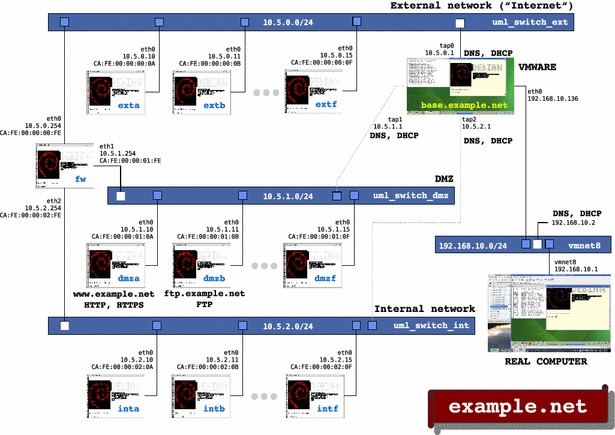
General structure of NETinVM. Virtual machines and networks within NETinVM

NETinVM is a VMware virtual machine image that includes, ready to run, a computer network of User-Mode Linux (UML) virtual machines. When started, the UML machines form a computer network named “example.net” whose general structure is shown in Fig. 1. This section, describes these three basic elements (the VMware virtual machine, the UML virtual machines and the virtual network) and how some critical infrastructure issues have been solved. For a detailed description, the NETinVM web page can be consulted (Pérez and Pérez 2014).

### VMware virtual machine image

The VMware virtual machine, named *Base*, provides the base to run and monitor the UML virtual machines, and its fully qualified domain name is “base.example.net”. *Base* includes 1 32-bit processor, 2 GB of RAM, a 20 GB SCSI hard disk, a DVD player, 1 network interface connected to VMware’s NAT network, USB controller, 1 sound card, and 1 graphics card. On this virtual hardware, version 12.1 of openSUSE (Novell 2008) is executed, which provides the KDE desktop, LibreOffice and C/C++ development tools. *Base* also includes the tools needed to monitor the execution of UML machines, such as Tcpdump or Wireshark. Obviously, it also includes UML and the disk image used by the UML virtual machines that will run in it. Even with all these tools installed, *Base* has around 13 GB of free disk space. This storage capacity allows to start and work with the UMLs, and also to install additional tools.

### UML virtual machines

The UML virtual machines (UMLs) are created using User-Mode Linux and, depending on the network they are connected to, they assume different roles: corporate workstation, internal server, router, bastion node, external server or Internet node. Each UML has the following virtual hardware: 1 32-bit processor, 128 MB RAM, 1 GB hard drive, and 1 network interface (except the UML that acts as a router—labeled as “fw” in Fig. 1, which has 3 interfaces). All UMLs use the copy-on-write technique provided by UML. Therefore, all of them initially start using the same file system, and each one writes his changes to a separate sparse file. In this file system the version 6 of Debian (2008) is installed, including appropriate tools for teaching networking, system administration and security topics. There are several advantages derived from all UML machines sharing the same root file system, which we call “reference file system” (RFS):

1. It saves space. Using copy-on-write, 19 UML machines can be running taking as little as 0.5 GB of *Base*’s disk.
2. It simplifies maintenance. Updating all UML machines with the latest security patches or adding a software package to all of them is as simple as doing it in one of them.
3. It simplifies its use. All UMLs are similar and have the same software installed.

### Virtual networks

NETinVM is pre-configured to create three interconnected virtual networks, playing the role of the corporate, perimeter and external networks of an organization. These networks are named “int” (for internal network), “dmz” (for DMZ or demilitarized zone, which is often used as a synonym for perimeter network) and “ext” (for external network). The networks are created using the “uml_switch” program included with UML. This program implements a virtual Ethernet hub or switch (configured as a hub in NETinVM). One of the UML machines, “fw” (for firewall), interconnects the three networks providing communication and packet filtering, as shown in Fig. 1. The rest of UMLs have a single network interface connected to the network they are named after, as follows (where X can be from “a” to “f”): *intX* UMLs are connected to the internal network. These machines only offer the SSH service. *dmzX* UMLs are connected to the perimeter network (DMZ). They are conceived as bastion nodes. In this network there are two machines with alias. “dmza” has the alias “www.example.net” and it provides HTTP and HTTPS services; “dmzb” has the alias “ftp.example.net” and it offers FTP. Finally, *extX* UMLs are connected to networks that are external to the organization (e.g., “Internet”). These three networks are connected through *base* to VMware’s “vmnet8” (NAT) virtual network, which allows the connection of UML to external (real) networks.

The default gateway for the internal and perimeter networks (machines “intX” and “dmzX”) is “fw”, the default gateway for “fw” is the IP address of “base” in the “ext” network, and the machines on the external network (“extX”) have “base” as the default gateway, and “fw” as the gateway to access the perimeter and internal networks. “fw” applies NAT to all traffic from the internal and perimeter networks that is going out through its external network interface, so that these packets get to the external network with 10.0.0.254 as source IP address. Therefore, the traffic among UML machines of the three networks always goes through “fw”, while the traffic directed to machines outside “base” goes through “fw” if and only if it comes from the internal or the perimeter networks. In any case, the traffic to the outside world always goes through “base”, which, as “fw”, has also enabled IP forwarding and NAT. Communications between “base” and any UML Machine are carried out directly, without passing through “fw” (provided that the IP of “base” corresponding to the network of the UML machine is used). This arrangement is convenient because it allows access from “base” to all UML machines using SSH, regardless of the configuration of routing and packet filtering in “fw”. The UML machines can communicate each other via standard network protocols. All UML machines have the SSH service enabled by default and there are bastion nodes offering HTTP and FTP services, but any other standard IP service can be also configured (NFS, SMTP, ...).

The configuration of SNAT in “fw” as described above is necessary so that responses to outgoing connections to Internet originated in the internal and perimeter networks get back through “fw”. If SNAT were not active in “fw”, the responses would be sent by “base” directly to the UML machines, thus bypassing “fw”.

### Inter-machine communication

The UML machines can communicate each other via standard network protocols. All UML machines have the SSH service enabled by default and there are bastion nodes offering HTTP and FTP services, but any other standard IP service can be also configured (NFS, SMTP, ...).

Communications between “base” and the UML machines can also be carried out through the network, with the advantage that “base” is directly connected to the three subnets and, therefore, it has access to all UML machines regardless of the configuration of “fw”.

Also, when a UML virtual machine starts, 3 virtual terminals appears in *Base*. In this way, the user can work with the UMLs even when the network is not operational, as if having physical access to the machines.

Finally, the UML machines have access to the directory “$HOME/uml/mntdirs/tmp” of *Base* using the path “/mnt/tmp”. To set up this correspondence, it is used UML’s “hostfs” file system. Thus, all of the UMLs and *Base* share a directory through which they can exchange information without network access.

### Configuration of UMLs

Although sharing the same reference file system (RFS) is very positive, it is clearly necessary that each UML virtual machine can be adapted to play different roles. For example, ‘fw’ has three network interfaces and performs packet filtering, ‘dmza’ provides HTTP and HTTPS, ‘exta’ only provides SSH, ...

The RFS includes one and only configuration tool, the script “configure.sh”, which is stored in “base” and is also accessible to the UMLs using the “hostfs” file system introduced before. When starting, every UML tries to run this script, whose algorithm is as follows:

1. Checks if the UML has already been configured. If so, it ends.
2. Marks the machine as configured.
3. Applies the default settings.
4. Applies the network specific settings.
5. Applies the machine specific settings.

The configuration (the default, network specific or machine specific) involves enabling services and/or execute orders. In any case, as the configuration is done only once per virtual machine, the changes have to be permanent and stored in the machine’s filesystem. For example, if a service “fw” is added, symbolic links must be added to “/etc/rcX.d” (where X is the default run level).

This configuration mechanism has three key advantages:

1. Configuration (even “configure.sh” itself) can be completely changed without starting any UML machine.
2. Once they are running (even after the first boot), UMLs have a standard Debian file system, since the only commands executed are those of the standard booting process.
3. Different configurations can be easily saved so that different exercises begin with a known different initial state.

### Backup and restore

NETinVM includes a tool for creating and restoring backups. To save the state of all of the UMLs is enough to run the script “uml_backup.sh”. And, to restore a previously saved state, it is just necessary to run the script “uml_restore.sh”. Both utilities use the standard KDE file dialog to select where to store copies (“uml_backup.sh”) and which backup to restore (“uml_restore.sh”). The only requirement is that the UMLs must be stopped to perform a backup or restoration.

Backups are TGZ files including configuration files (which are small) and copy-on-write files (which are sparse files that include only changes made with respect to the RFS). Thus, each backup usually takes some KB or, at most, a few MB of disk space. This makes it possible to perform dozens of exercises, each one with multiple restoration points, without consuming too much storage space.

## Results and discussion

NETinVM has been intensively used at University of Valencia since 2012 for teaching courses related to security, system administration and network planning. These courses are part of the degree curricula for Telematics Engineering and Computer Engineering and master curricula for web services, and they are based on different learning techniques: Problem-Based Learning (PBL) and traditional Lecture-Based Learning (LBL). Also, NETinVM has been used in other scenarios such as books and web-based courses. In this section, we analyze the use of NETinVM in all these environments.

### Lecture-based learning in a computer security course

Traditional Lecture-Based Learning, where the teacher makes an oral presentation intended to present the main concepts of the course, is usually complemented with exercises to be carried out by the students. This is the case for computer security, a mandatory course scheduled in the third year of both the Degree in Computer Engineering (DCE) the Degree in Telematics Engineering (DTE). This is an introductory course of computer security and thus it has a wide scope. Nevertheless, it has the goal of providing the students with practical skills. In order to achieve this goal, we have extended the traditional LBL model with the following teaching activities, made possible by NETinVM: demos, exercises and labs. *Demos*, are practical explanations where the teacher performs and discusses the activity with the students in a lecture session. This kind of activity provides the students with deeper insights and it fosters their participation. NETinVM allows the students to reproduce later the same activities or even test new cases. *Exercises* consist of practical assignments involving several hosts and networks that students must do autonomously. By using NETinVM, these activities can be securely performed in a realistic and reproducible scenario. Finally, *labs* are guided sessions where complex exercises are performed by the students under the teacher supervision. NETinVM allows the students to complement the guided session with further optional work. A representative example of a *demo* could be using Snort as a NIDS. This demo consists of running the Snort intrusion detection software (Snor_team 2014), and showing how alerts are generated when suspicious activities are detected. The examples used were scanning the network with Nmap, connecting as administrator to a remote SQL database, and asking the DNS server for a zone transfer. While performing these activities, the network traffic was captured with Wireshark and the results were discussed with the students. An example of *exercise* carried out in the classroom is understanding security alerts. Two CVE alerts were selected, and the students were asked to test if “base” or the UML machines were vulnerable, and if there was an exploit that worked against them. Finally, a representative example of *labs* is firewall configuration. Using Linux Iptables, the lab goes from configuring a single machine (personal firewall) to configuring a machine which is responsible for the interconnection and filtering of the three NETinVM networks, thus providing a real case scenario. The lab includes both basic static rules and more advanced possibilities as packet logging or stateful rules.

Next, we describe some representative examples of these teaching activities carried out during the 2013–2014 year. Two of the demos performed were the following ones:

- *Public key cryptography in SSH for server authentication* In this demo, an initial connection to a SSH server is started. Since the server’s public key is not present in the client’s known hosts file, a confirmation message appears. The importance of answering this question is discussed with the students, highlighting that this verification is the only protection against man-in-the-middle attacks.
- *Using Snort as a NIDS* This demo consists of running the Snort intrusion detection software (Snor_team 2014), and showing how alerts are generated when suspicious activities are detected. The examples used were scanning the network with Nmap, connecting as administrator to a remote SQL database, and asking the DNS server for a zone transfer. While performing these activities, the network traffic was captured with Wireshark and the results were discussed with the students.

Two examples of the exercises proposed were the following ones:

- *Understanding security alerts* Two CVE alerts were selected, and the students were asked to test if “base” or the UML machines were vulnerable, and if there was an exploit that worked against them.
- *Analysis of Snort rules* Students were asked to perform two kinds of remote access to a database. Each access should trigger a snort alert. They had to capture network activity, correlate the information in the captured packets with the corresponding snort rule, and justify why the alert was or was not generated, depending on the case. This exercise is an extension of the second example demo explained above. In this way, once the session in the classroom finishes, the students can not only reproduce the demo by their own, but they can also extend that demo through this exercise.

Finally, these are two examples of the labs carried out:

- *Firewall configuration* Using Linux Iptables, the lab goes from configuring a single machine (personal firewall) to configuring a machine which is responsible for the interconnection and filtering of the three NETinVM networks, thus providing a real case scenario. The lab includes both basic static rules and more advanced possibilities as packet logging or stateful rules.
- *Forensic analysis* Students are challenged to use The Sleuth Kit (TSK) and Autopsy tools (Carrier 2014) to construct a time line and retrieve information from a file system image of a hacked UML machine. They have previously learned to obtain file system images in a demo in the classroom. Similarly, another demos have been performed to introduce them to the TSK and Autopsy tools. The challenge includes finding a binary trojan, recovering deleted files related to malicious activity, and finding hidden information in the file system.

It must be noticed that NETinVM permits to easily modify a given activity to become a different kind of activity in a different year. This is possible because the same platform (NETinVM) is used for all three kind of activities, and this platform is available for the students anywhere and anytime. For example, it is easy to change one demo into one or more autonomous exercises. Also, it is easy to convert a lab session into a set of demos or exercises.

We have qualitatively and quantitatively evaluated the approach followed in this course. The quantitative evaluation comes from numeric evaluations of the course carried out by the students as part of the University of Valencia’s quality assessment protocol. This protocol includes anonymous annual surveys with questions regarding different aspects of the teaching-learning process. The most significant one for our work is the evaluation of the methodology, but we have also included the global average for the course, since it is a global assessment of both the NETinVM tool and its use throughout the course. Numeric values can be between 0 and 5, with a mark of 5 being the best possible score. Table 1 shows the quantitative evaluation of the course made by the students. The first row in this table shows the specific results for the methodology followed in the security course, and the second one shows the general results for the course. The first (most-left) column shows the results for the security course in the Degree in Computer Engineering, and for comparison purposes the second column shows the average values obtained in all the courses of this Degree. The two next columns show the analog values for the Degree in Telematics Engineering, and the last column, labelled as “Univ.”, shows the average values obtained by all the courses taught in the University of Valencia. This table shows that the marks obtained by the security course in both degrees are significantly higher than the average values of their degrees and the University. These values clearly show that the students greatly appreciate the approach followed by the course, that NETinVM has made possible.

**Table 1:** Students course evaluation

	Security (DCE)	DCE	Security (DTE)	DTE	Univ.
Methodology	4.49	3.63	4.04	3.74	3.88
Course average	4.48	3.52	4.08	3.67	3.83

Data from University of Valencia’s quality assessment protocol

In order to complement this evaluation, we have used a reduced version of the Critical Incident Questionnaire, proposed by Brookfield (2014a). We have asked the students to write down the best and the worst things about the course. Although they were not specifically asked about the utilization of NETinVM, their comments clearly show that they appreciate the practical approach made possible by this tool. Effectively, the most repeated positive opinions were (in descending order) the following ones: excellent demos; up to date and interesting content; agile and enjoyable classes; excellent laboratory assignments, and Lab assignments closely related to theoretical contents.

These comments clearly show that using NETinVM throughout the course, and the practical activities that can thus be added to the traditional LBL, are greatly appreciated by the students.

### Problem based learning in a network planning course

Problem-based learning (PBL) (Barrows and Tamblyn 1980; Savery 2006) is a teaching methodology where the student’s learning process relies on a problem (constructed by the teacher or other students) similar to those problems that the student will face in real life. The teacher is limited to be a “coach” or a moderator, instead of the source of knowledge, while the students should collaboratively solve the problem through cooperative learning. PBL methodology was applied in the context of a network planning course in the Engineering School, at University of Valencia. This is a mandatory course scheduled in the fourth year of the Degree of Telematics Engineering. The course focuses on network planning and management, including saturation and bottleneck detection. Concretely, NETinVM has been used to design a lab session where practical ways of detecting network saturation should be learned through PBL methodology.

The problem is set up as a team contest for winning the Best Hacker and the Best Administrator Awards. Each team should design and implement a secret procedure that tries to saturate the NETinVM networks. The only rule is that the saturation procedure must not require to become root in any of the NETinVM hosts. As a previous work to the lab session (prior to the contest), each team should design, implement and try as many different procedures they want in order to saturate the networks in NETinVM, and they can demand help to the teacher to guide the process. Prior to the start of the contest, each team should privately present the teacher (the saturation procedure is secret for the rest of the teams) a written report describing the final procedure they have implemented. The awards are based on a single real-time competition that takes place in one or more lab sessions, with as many rounds as participating teams. When it is the turn for each team, that teams becomes the hacker in that round, and the team components should implement the saturation procedure designed by that team in the NETinVM copies of the rest of the teams. The rest of the teams act as administrators in that round, and they should detect the source node (the NETinVM host) and the program(s) causing the network saturation as soon as possible, within maximum time of 20 min. Any erroneous detection is “punished” with the rating of that team as the last one in that round. All the rounds are timed, starting when every team (except the one acting as the hacker) has its NETinVM network saturated, and finishing either when all the teams have found the origin of the network saturation, or when 20 min have passed. After the contest, there is a round table discussion where all the teams present their saturation procedure to the rest of the teams, as well as the strategy and commands/programs used for detecting the origin of the saturation. Since the exercise has not a limited number of solutions, the validity, advantages and disadvantages of each proposal are discussed. The teams are marked in each round as both administrators and hackers. As administrators, the teams are marked according to the time required for finding the cause of the network saturation (in inverse order). As hackers, they are marked according to the time took by the first team that discovered the origin of the saturation (the longer time, the higher they are marked). The aggregated marks for all the rounds will determine the final team rankings for both contests, being the winner of each contest the team heading the ranking. The participation in the contests ensures a minimum mark, but the position in each ranking determines the mark as each of the roles. The final mark obtained by each team is the in the average value of the mark obtained in the two contests. The prize for each contest winner is some additional mark, ranging in 0.5 and 1 points out of 10.

The final resolution activity took two lab sessions (there were five teams, each one composed of four members), and the students reported an average dedication of 5 h per team member to the particular problem resolution, including team meetings (80 % of time) and individual work (20 %). All the groups showed great interest in the activity, and they developed sophisticated problem solutions showing a deep knowledge of Linux and network fundamentals. No erroneous detections happened in the contest, and one team achieved that the rest of the teams except one exceeded the maximum time to find the origin of the saturation.

The feasibility of the proposed PBL activity fully relies on NETinVM, since the saturation of any network should significantly affect the actual network infrastructure. Therefore, we asked the students to evaluate the activity, instead of the tool. Concretely, we made an anonymous survey, asking the students (grouped by teams) to evaluate the proposed activity in regard to standard lab sessions where students should perform practical exercises following the guide notes provided by the teacher. A mark of 5 out of 10 corresponds to an evaluation where the students equally value both kinds of lab sessions, a mark of 0 means that they absolutely prefer the standard lab sessions, and a mark of 10 means that they definitely prefer the activity based on PBL methodology. We also asked to report the main feature(s) of the activity that they liked the best. Table 2 shows the results of the survey. This table shows that the students significantly prefer the proposed activity. Also, they valued the freedom for designing any feasible solution and the format of contest among the existing teams as the best two aspects of the activity (in that order). The first aspect would not be possible without the use of NETinVM, since it provides the students with a virtual copy of real networks and hosts, allowing them to test any solution. Therefore, these results validate NETinVM as a valuable tool for activities based on PBL methodology.

**Table 2:** Evaluation of the activity provided by the students

	Teams					Avg.
	1	2	3	4	5	
Marks	8.0	9.0	7.0	8.5	8.0	8.1

### Using NETinVM for teaching enterprise web applications development

Enterprise web applications are built by integrating specialized components (web servers, application servers, database management systems, ...) connected via networks. At postgraduate level, students must be able to develop skills in integrating all of these components in real-world scenarios. This is the case of the Master in Systems and Services in the Information Society, where a common platform for all the courses of the master was desirable. The authors engaged in the project of adapting NETinVM to provide a satisfactory teaching and learning environment for enterprise web application development, including facets such as application development, application deployment, server administration and security.

The solution consisted of adapting the standard configuration of NETinVM to suit the specific needs of this project. The following changes were performed: Installing and configuring an application server (Glassfish) in “dmzc”; installing and configuring MySQL and LDAP in “intb”; installing and configuring Eclipse in “base”; Adapting the rules at “fw” to the new environment. In particular, the application’s server front-end interface (port 80) had to be publicly accessible, the application’s server administrative interface had to be accessible only from selected nodes of the internal network, and the applications’ server should be able to contact the LDAP and MySQL internal server.

This adapted version of NETinVM provided master’s students and teachers with a common platform that proved to be appropriate to conduct all the practical exercises and demonstrations, with the following advantages (Pérez et al. 2011): the students had to learn only a single tool (NETinVM) that was shared by different subjects in different areas, such as operating system administration, computer and network security, and web development; students were able to develop, deploy and test their applications in their own portable environment without compromising real systems or networks; students and teachers shared a common environment, so classroom demonstrations could be reproduced by students; finally, using the same tool throughout the master allowed for better coordination among teachers of different subjects.

### Other uses of NETinVM

The ease of portability and reproducibility of a realistic scenario yielded by NETinVM make this virtual machine an ideal tool for Massive Open Online Courses (MOOC). In this way, it has been used as the platform for a new Massive Open Online Course (MOOC) at University of Valencia (Pérez 2016). In this open course, the networks and virtual machines included in NETinVM are used for providing each student with its own virtual lab where practical network and security exercises can be performed.

Nevertheless, NETinVM has been successfully used in other scenarios by people not related to the University of Valencia. Effectively, in the book “CASP: CompTIA® Advanced Security Practitioner, Study Guide”, by Gregg (2012), the author uses NETinVM in 11 out of 20 labs. These labs provide a hands-on approach necessary to fully understand the concepts introduced in the book, which is preparatory to the “CompTIA® Advanced Security Practitioner” exam (Brookfield 2014b). NETinVM is used for labs such as port scanning, network traffic analysis, web vulnerability assessment, system auditing, network intrusion detection, or rootkit detection.

Another example of use is the paper titled “Using OSSEC with NETinVM” (Allen 2010), submitted by Jon Mark Allen as part of the GIAC (GCIH) Gold Certification from the SANS Institute (2014). This paper, presented in September 17, 2010, uses NETinVM as an appropriate virtual scenario for installing and customizing the host-based intrusion detection system OSSEC (2014). Using NETinVM allowed the author to configure OSSEC to comply with a security policy. In addition, it also made possible launching attacks, checking that alerts were effectively generated, and seeing how OSSEC automatically responded to the attacks.

Finally, NETinVM has also been adapted to suit more specific requirements. This is the case of the “Lab in a box” of the PenTestlaboratory, where NETinVM was modified to build a virtual laboratory for penetration testing courses (PenTestlaboratory 2014). In this set up, UML machines where specifically configured to be vulnerable, in order to become potential targets of pentesters.

## Conclusions

This paper has proposed NETinVM, a tool based on nested virtualization that includes a fully functional lab in a single virtual machine. Also, it has analyzed and evaluated how it has been used in different environments. The results show that this tool makes it possible to perform demos, labs and practical exercises, greatly appreciated by the students, that would otherwise be unfeasible. In addition, it allows to reproduce the results of the proposed exercises, providing portability and allowing the students to work autonomously. Also, NETinVM has been adapted to suit other scenarios, such as enterprise web application development or penetration testing.

As a future work, the authors plan to add support for controlled remote access, thus allowing the instructor to provide students with remote assistance.

## Availability and requirements

- Project name: NETinVM
- Project home page: http://www.netinvm.org
- Hardware requirements:
  - Processor with hardware support for virtualization
  - 4 GB RAM
  - 20 GB of available hard disk space
- Software requirements:
  - VMware Player, VMware Workstation or Virtualbox
  - Operating system(s): Any of the OS on which VMware or Virtualbox works.
